# Hemorrhage After Endovascular Recanalization in Acute Stroke: Lesion Extent, Collaterals and Degree of Ischemic Water Uptake Mediate Tissue Vulnerability

**DOI:** 10.3389/fneur.2019.00569

**Published:** 2019-06-04

**Authors:** Jawed Nawabi, Helge Kniep, Gerhard Schön, Fabian Flottmann, Hannes Leischner, Reza Kabiri, Peter Sporns, André Kemmling, Götz Thomalla, Jens Fiehler, Gabriel Broocks, Uta Hanning

**Affiliations:** ^1^Department of Diagnostic and Interventional Neuroradiology, University Medical Center Hamburg-Eppendorf, Hamburg, Germany; ^2^Institute of Medical Biometry and Epidemiology, University Medical Center Hamburg-Eppendorf, Hamburg, Germany; ^3^Department of Radiology, University Hospital Münster, Münster, Germany; ^4^Department of Neurology, University Medical Center Hamburg-Eppendorf, Hamburg, Germany

**Keywords:** ischemic stroke, intracerebral hemorrhage, outcome prediction, computed tomography, thrombectomy, collateral score, ischemic brain edema, ASPECTS

## Abstract

**Background and Purpose:** Intracranial hemorrhage (ICH) remains a major complication of endovascular treatment (ET) in acute stroke. The aim of this study was to identify clinical and imaging predictors for ICH in patients with acute ischemic stroke undergoing successful ET.

**Methods:** We performed a retrospective analysis of patients with large vessel occlusion in the anterior circulation who underwent successful ET at our university medical center between 2015 and 2018. ICH was diagnosed on non-enhanced CT and a binary outcome was defined: ICH occurrence in the immediate post-interventional phase within 12–36 h (yes/no). The impacts of clinical, radiological, and interventional parameters on outcome were assessed in logistic regression models.

**Results:** One hundred and seven patients fulfilled the inclusion criteria. 37 (34.6%) showed an ICH of which 7 (6.5%) patients were diagnosed as symptomatic and 30 (28.04%) as asymptomatic. Multivariable regression analyses identified a lower ASPECTS (adjusted odds ratio (OR) 1.95, 95%CI: 1.4–3.63, *P* = 0.037), low collateral score (adjusted OR 0.12, 95%CI: 0.03–0.49, *P* = 0.003) and high Net Water Uptake (NWU) (adjusted OR 1.56, 95%CI: 2.34–1.03, *P* = 0.007) as independent predictors of ICH after successful ET.

**Conclusions:** CT-based quantitative NWU, ASPECTS, and collateral score mediate tissue vulnerability and are reliable independent predictors of a bleeding event after successful ET. This imaging-based prediction model might be useful for early stratification of patients at high risk of a bleeding event after ET, especially with low ASPECTS.

## Introduction

Large randomized controlled trials have demonstrated efficacy of endovascular treatment (ET) over medical treatment in patients with acute ischemic stroke (AIS) (1, 2). Despite this treatment success, intracranial hemorrhage (ICH) remains a major complication (1). Evaluating the risk of ICH becomes an important issue for continuously improving the efficacy of this new treatment strategy. In the HERMES meta-analysis, 4.4% of patients suffered from symptomatic ICH (SICH) (1). ICH is classified as a symptomatic bleeding according to clinical relevance and implies a clear causal relationship between clinical deterioration and bleeding event. Fatal clinical outcomes are reported in 41.4% cases of SICH (3, 4).

A new meta-analysis observed that ET increases the risk of any ICH compared with medical treatment, when rates of asymptomatic ICH (AICH) after ET are also considered (5). This increased risk was mainly related to cases with AICH after ET. At the same time, in contrast to the labeling, AICH may not be asymptomatic, as recently AICH has been associated with a decreased likelihood of excellent outcome after ET (6).

Imaging markers of ischemic damage may influence the rates of a bleeding event as early ischemic changes seen on non-contrast CT are strongly associated with higher rates of ICH (5).

However, no clearly described guidelines exist for patients at high risk for a bleeding event after ET and might be important to evaluate for continuously improving this treatment strategy. Imaging-based risk factors may provide an early and feasible stratification of high-risk patients for a bleeding event and improve clinical outcome. We hypothesized that imaging markers can be used to predict the respective bleeding event after successful ET. We therefore performed a retrospective study using the widely accepted Heidelberg Bleeding Classification (7), to identify independent risk factors for a bleeding event after successful ET in patients with AIS.

## Methods

### Study Population

We retrospectively studied all ischemic stroke patients with large vessel occlusion between June 2015 and March 2018 in our university hospital, which is a high-volume tertiary stroke center.

As inclusion, we defined: (1) evidence of large vessel occlusion of the middle cerebral artery (MCA) or terminal internal carotid artery (ICA); (2) initially performed multimodal CT protocol with CT angiography (CTA) and perfusion CT (CTP) and follow-up CT admitted 24 h after imaging; (3) thrombolysis in cerebral infarction scale (TICI) 2b or 3; (4) known time from symptom onset to imaging <6 h; (5) National Institute of Health Stroke scale (NIHSS) score above 3; (6) documented NIHSS after 24 h and modified Ranking Scale (mRS) after 90 days; (7) absence of a preexisting thromboembolic or hemodynamic infarctions in admission non-enhanced CT (NECT). Patients were excluded for the following reasons: (1) occlusion in posterior circulation, (2) recanalization with TICI 0–2a, (3) inadequate clinical documentation or missing patient consent, (4) contrast extravasation, (5) ICH due to mechanical vessel perforation and (6) inadequate image acquisition. Baseline patient characteristics were retrieved from medical records, including modified Rankin Scale (mRS) after 90 days. ASPECTS rating was performed by an experienced neuroradiologist and verified by an attending neuroradiologist. Discrepancies about the ASPECTS were settled by joint discussion of the two readers. Recanalization status was derived for every patient: (a) vessel recanalization after successful thrombectomy (TICI 2b or 3); (b) persistent large vessel occlusion in patients with failed recanalization after endovascular thrombectomy (TICI 0–2a) or patients who did not receive ET. The neuroradiologist who performed the intra-arterial procedure documented the TICI scores with subsequent verification by a second attending neuroradiologist. Inaccuracies were corrected in consensus reading, if necessary. Data were analyzed after ethical board approval, and informed consent was waived by the institutional review board. The data that support the findings of this study are available from the corresponding author upon reasonable request.

## Image Acquisitions

All CT scans were performed on a 256 slice scanners (Philips iCT 256) with the following imaging parameters: NECT with 120 kV, 280–320 mA, 5.0 mm slice reconstruction; CTA: 100–120 kV, 260–300 mA, 1.0 mm slice reconstruction, 5 mm MIP reconstruction with 1 mm increment, 0.6 mm collimation, 0.8 pitch, H20f soft kernel, 80 mL highly iodinated contrast medium and 50 mL NaCl flush at 4 mL/s; scan starts 6 s after bolus tracking at the level of the ascending aorta). CTP: 80 kV, 200–250 mA, 5 mm slice reconstruction (max. 10 mm), slice sampling rate 1.50 s (min. 1.33 s), scan time 45 s (max. 60 s), biphasic injection with 30 ml (max. 40 ml) of highly iodinated contrast medium with 350 mg iodine/ml (max. 400 mg/ml) injected with at least 4 ml/s (max. 6 ml/s) followed by 30 ml sodium chloride chaser bolus. All perfusion datasets were inspected for quality and excluded in case of severe motion artifacts.

## Image Analysis

### Collateral Score Classification, Quantification of Ischemic Brain Edema and Intracerebral Hemorrhage Classification

The following image analysis was performed on anonymized CT imaging material and evaluated independently by two radiologists with 2 and 6 years of dedicated neuroradiology experience, blinded to all clinical and imaging information except stroke side.

### Collateral Score

**Figure 1:**
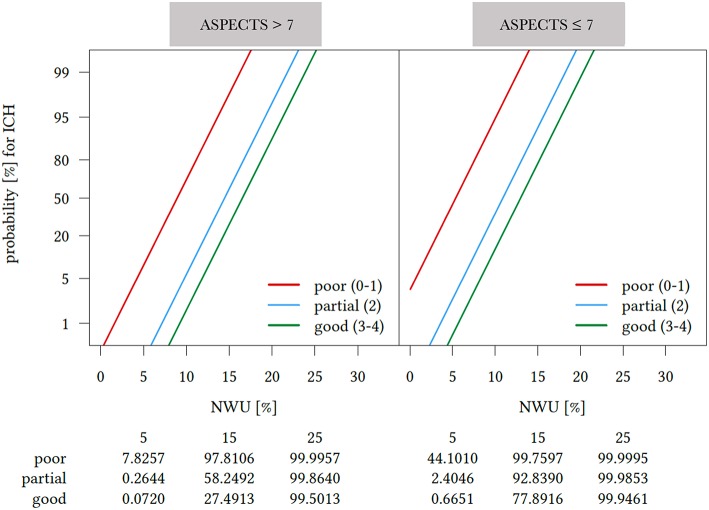
Probability of intracerebral hemorrhage (ICH) after successful endovascular treatment by collateral score, early Net Water Uptake (NWU) and ASPECTS after logistic regression analysis adjusted for age and sex. Impact of Net Water Uptake (NWU) (x-axis) and Alberta stroke program early CT score (ASPECTS), dichotomized for ASPECTS ≤7 and ASPECTS >7, and collateral score (straight lines) on the probability of intracerebral hemorrhage (ICH) after successful recanalization (y-axis) based on multivarible linear regression analysis adjusted for sex and age. For any given early NWU, the probability for developing ICH was modulated by the level of collateral score and ASPECTS. The probability for ICH was higher for patients with poor collateral status, higher NWU. Colored lines indicate collateral scores [good (3–4); partial (2); and poor (3–4)]. Collateral Scores were grouped into good, partial, and poor collaterals (11). Dichotomization of ASPECTS was performed according to Cut-off Value in ROC-Analysis. ASPECTS, Alberta stroke program early CT score; ICH, Intracerebral hemorrhage; NWU, Net Water Uptake.

CT angiography (CTA) collaterals was assessed independently on admission intracranial CTA MIPs and scored according to the grading system of Souza et al. (8) into grades 0 to 4: CS: 0 = absent collaterals in >50% of an MCA M2 branch (superior or inferior division) territory; 1 = diminished collaterals in >50% of an MCA M2 branch territory; 2 = diminished collaterals in <50% of an MCA M2 branch territory; 3 = collaterals equal to the contralateral hemisphere; and 4 = increased collaterals. The grading system of Souza et al. used volume threshold that respects precisely the territory of the terminal ICA and MCA (8). Clinical studies have supported the clinical importance of this new volume threshold (9, 10). For the sake of clarity in Figure 1, collateral grades were grouped according to current state of literature into good (collateral score 3–4), partial (collateral score 2), and poor (collateral score 0–1) (11).

### Quantification of Ischemic Brain Edema

Ischemic brain edema was quantified in admission and follow-up CT using quantitative lesion water uptake (NWU). The edematous proportion of the infarct lesion (%-water uptake) can be quantified using CT-densitometry as reported elsewhere [22, 23, 35]. Therefore, the anonymized admission CT imaging material was segmented manually using commercially available software (Analyze 11.0, Biomedical Imaging Resource, Mayo Clinic, Rochester, MN) to derive net water uptake (NWU).

### Intracerebral Hemorrhage Classification

The follow-up CT within 12–36 h after ET was screened for secondary ICH. Diagnosis and classification of bleedings events was performed according to the Heidelberg Bleeding Classification (7) and found in detail in the supplemental. In brief, bleeding events were assigned to the subtypes of hemorrhagic infarction (HI) 1, HI 2, parenchymal hematoma (PH) 1, PH 2, remote PH, intraventricular hemorrhage, subarachnoid hemorrhage, or subdural hemorrhage. Secondly, stepwise relatedness of neurological deterioration and imaging findings was performed for the classification of patients with SICH and AICH (7). Hyperdense phenomena without mass effect on CT and disappearance with 24 h on follow-up CT were classified as contrast extravasation (12).

## Statistical Analysis

Kolmogorov-smirnov tests were used to determine if the data sets were well-modeled by a normal distribution. For categorical data, absolute and relative frequencies are given. Univariable distribution of metric variables is described by median and interquartile range (IQR). Patients without ICH vs. with ICH were compared by Mann-Whitney *U*-test for metric outcome variables and by chi-square test for categorical outcome variables. Interrater reliability was measured with kappa statistic and calculating Cohen's k.

Logistic regression analysis was performed to assess the association between the clinical and radiological parameters and an ICH occurrence in the immediate post-interventional phase after successful ET (yes/no). Multivariable model building was performed using a stepwise variable selection procedure: in a first step, all factors were fitted together by a stepwise forward selection (inclusion: *P* value of the score test ≤0.05 and exclusion: *P* value of the likelihood ratio test >0.1). Then, the factors of the model from step 1 were fitted together with all pairwise interactions in a second block using stepwise forward selection (inclusion: *P* value of the score test ≤0.05 and exclusion: *P-*value of the likelihood ratio test >0.1). Given for selected variables are odds ratios with 95% confidence interval (CI) and *P* value of likelihood ratio test. For nonselected variables, *P* value of score test is displayed. The diagnostic performance to predict ICH and also SICH after successful ET was assessed by ROC analysis with increasing discrimination thresholds for clinical and radiological parameters (Supplemental Material). Local, unadjusted *P* < 0.05 were considered as statistically noticeable. Analyses were performed using SPSS version 25 (IBM Corporation, Armonk NY) and R (R Core Team. R: A Language and Environment for Statistical Computing. R Foundation for Statistical Computing. Vienna, Austria, 2017).

## Results

### Baseline Characteristics

Out of 336 patients with AIS who underwent ET, 107 fulfilled the inclusion criteria. Among other exclusion criteria, 52 patients had an occlusion in the posterior circulation and 119 patients a TICI 0–2a (Supplemental Figure 1). As shown in Table 1, median age in patients with ICH was 79 years (IQR: 69–84) with 21% being women vs. 75 years (IQR: 64.8–80.0), and 34% women without ICH. 63 patients (58.9%) received intravenous thrombolysis before endovascular therapy. Patients with ICH after successful ET had a significantly higher rate of atrial fibrillation (27.1 vs. 36.4%, *P* = 0.006). Also, baseline Alberta Stroke Program Early CT (ASPECTS) score [median 7 (IQR: 5.5–8) vs. 8 (IQR: 6–9), *p* = 0.03] and collaterals score [median 1 (IQR: 1.2) vs. 2 (IQR: 2.3), *P* < 0.001] was significantly lower, with a higher NWU [median 13% (IQR: 10–16%) vs. 5% (IQR: 3–9%), *P* < 0.001] in patients with ICH. Interobserver agreement in the collateral score classification was good (Cohen's Kappa: 0.91; 95% CI 0.86–0.95; *P* < 0.001). Also, admission NIHSS differed significantly, with a higher NIHSS on admission, after 24 h and at discharge in patients with ICH [median 19 (IQR: 16–20) vs. 15 (12–18), *P* = 0.005; median 19 (IQR: 13–34.5) vs. 10 (IQR: 4/16), *P* < 0.001; median 14 (IQR: 7.5–18.5) vs. 5 (IQR: 1–9.8), *P* < 0.001, respectively] in patients with ICH.

**Table 1:** Comparison of baseline demographic, clinical and radiological characteristics between patients with intracerebral hemorrhage and those with no intracerebral hemorrhage.

**Baseline characteristics**	**All (*n* = 107)**	**Without ICH (*n* = 70)**	**With ICH (*n* = 37)**	***P-V*alue**
Age [years], median (IQR)	76 (65.0; 81.0)	75 (64.8; 80.0)	79 (69; 84)	0.074
Female, *n* (%)	55 (51.4)	34 (48.6)	21 (56.8)	0.420
Hypertension, *n* (%)	60 (56.1)	36 (51.4)	24 (64.9)	0.183
Diabetes mellitus, *n* (%)	20 (18.7)	14 (20.0)	6 (16.2)	0.633
Atrial fibrillation, *n* (%)	39 (36.4)	19 (27.1)	20 (54.1)	0.006
Smoking, *n* (%)	15 (16.9)	10 (18.2)	5 (14.7)	0.670
Dyslipidemia, *n* (%)	23 (21.5)	16 (22.9)	7 (18.9)	0.637
**CT PARAMETERS, MEDIAN (IQR)**				
Aspects	8 (6;9)	8 (6.0;9.0)	7 (5.5; 8.0)	0.030
Collateral Score	2 (1.0;3.0)	2(2.0; 3.0)	1 (1.0;2.0)	<0.001
Net water uptake (NWU)	0.04 (0.00; 0.1)	0.05 (0.03; 0.09)	0.13 (0.1; 0.16)	<0.001
Stroke Cause, *n* (%)				0.054
Cardioembolic	56 (52.3)	33 (47.1)	23 (62.2)	
Large-artery atherosclerosis	42 (39.3)	28 (40.0)	14 (37.8)	
Others	9 (8.4)	9 (12.9)	0 (0)	
**PROCEDURE PROCESS AND RESULTS**				
General anesthesia, *n* (%)	75.0 (70.1)	52 (74.3)	14 (37.8)	0.193
Intravenous thrombolysis, *n* (%)	63 (58.9)	26 (37.1)	19 (51.4)	0.250
OTI [h], median (IQR)	2:35 (1:06; 3:44)	2:24 (0:59; 3:42)	2:40 (2:07; 3:51)	0.438
ITR [h], median (IQR)	1:47 (1:23; 2:03)	1:40 (1:19; 1:58)	1:53 (1:33; 2:10)	0.243
Passes of retriever	2 (1;2)	2 (1;2)	2 (1;3)	0.071
mTICI, (2b, 3 grouped), *n* (%)	107 (100)	70 (100)	37 (100)	0.486
**CLINICAL PARAMETERS**				
NIHSS on admission	13 (12;19)	15 (12.0; 18.0)	19 (16.0;20.0)	0.005
NIHSS after 24 h	3 (5;20)	10 (4.0; 16.0)	19 (13.0; 34.5)	<0.001
NIHSS at discharge	1.5 (3;14)	5 (1.0; 9.8)	14 (7.5; 18.5)	<0.001
90 days mRS, median (IQR)	4 (3.2; 4.0)	3(2.5; 3.6)	5 (4.3; 5.3)	<0.001
0–1, *n (%)*	28 (28.0)	25 (37.3)	3 (9.1)	0.003
2–3, *n (%)*	15 (15)	11 (16.4)	4 (12.1)	0.572
4–6, *n (%)*	55 (51.4)	29 (44.6)	26 (78.8)	0.001

*ICH, intracerebral hemorrhage; IQR, interquartile range; TICI, thrombolysis in cerebral infarction; NIHSS, National Institutes of Health Stroke Scale; TICI, thrombolysis in cerebral infarction; INR, international normalized Ratio*.

### Intracerebral Hemorrhage Classification

Intracranial hemorrhage (ICH) was identified in 37 (34.6%) patients vs. 70 (65.4%) without ICH within 24 h after ET on follow-up CT. After establishing the relatedness of neurological deterioration and imaging findings, seven bleeding events (6.5%) were classified as SICH and 30 (28.04%) as AICH (Supplemental Table 1) (7). Distribution of the bleeding events are given in detail in the Supplemental Table 2. Contrast extravasation was observed in seven patients (*n* = 7) (12). Interobserver agreement in the evaluation of bleeding classification was good (Cohen's Kappa coefficient: 0.87; 95% CI 0.78–0.90; *p* < 0.001).

### Predictors of ICH After Successful ET

Logistic regression analysis was performed to assess the association between various clinical and radiological parameters and the incident of ICH after successful ET. In univariable logistic regression, higher NWU (*P* < 0.001), lower collateral score (*P* < 0.001), lower ASPECTS (*P* = 0.041), and higher NIHSS (*P* = 0.027) on admission were associated with ICH after successful ET (Supplemental Table 3). Multivariable logistic regression analysis identified higher rates of NWU (adjusted OR 1.56 per median percentage, *P* = 0.007), a lower collateral score (adjusted OR 0.090 per median, *P* = 0.008), and lower ASPECTS (adjusted OR 1.95 per median, *P* = 0.037) as independent predictors of ICH after successful ET (Table 2; Supplemental Table 4). For the sake of clarity dichotomization of ASPECTS was performed in the effect plot (Figure 1), according to the cut-off in ROC analysis. Differences in NWU had the biggest impact on the risk of ICH in patients with good and partial collaterals, especially between 5 and 15% NWU. Above a NWU of 15% the risk for ICH accumulated over 90%, independently from ASPECTS and collateral score. From a different viewpoint, the risk for ICH increases gradually with lower collateral status and higher NWU. In patients with ASPECTS <7 the overall risk of an ICH was higher compared to patients with ASPECTS >7 (Figure 1).

**Table 2:** Multivariable analysis of predictors of intracerebral hemorrhage after recanalization.

	**OR**	**95%CI**	***P-*Value**
Aspects	1.95	1.40–3.63	0.037
Collateral score	0.12	0.03–0.49	0.003
Net water uptake (NWU)	1.56	2.34–1.03	0.007

*Non-significant independent variables are not displayed (age, sex, arterial hypertension, diabetes mellitus, atrial fibrillation, smoking, dyslipidemia, intravenous thrombolysis, NIHSS on admission)*.

*Given for selected variables are Odds Ratios (OR) with 95% confidence interval (CI) and p-value of likelihood ratio test*.

### Association of ICH After Successful ET and Functional Clinical Outcome

**Figure 2:**
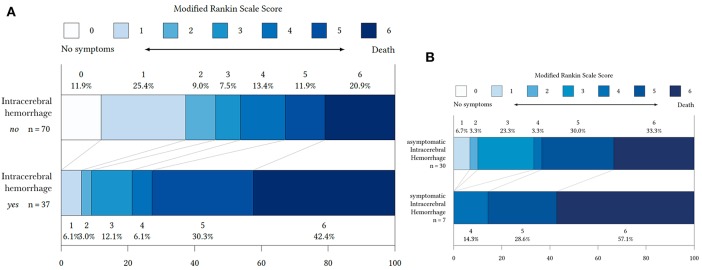
Distribution of modified Rankin Scale (mRS) at 90 days in patients with and without ICH **(A)** and symptomatic ICH and asymptomatic ICH **(B)**.

Patients with ICH had a significantly worse clinical outcome with a median of 5 (IQR: 4.3–5-3) vs. 3 (IQR: 2.5–3.6) in patients without ICH (*P* < 0.001). In detail, unfavorable clinical outcome at 90 days (mRS 4–6) occurred more often in patients with ICH (78 vs. 44.6%, *P* < 0.001). The proportions of patients with excellent clinical outcome (mRS 0–1) at 90 days was lower in patients with ICH (78.8 vs. 44.6%, *P* = 0.003) (Table 1). Subgroup analysis of patients with ICH demonstrated that patients with SICH had only unfavorable clinical outcomes at 90 days (mRS 4–6). In contrast, patients with AICH had less patients with fatal outcome (mRS 6 33.3% in AICH vs. 56.1% in SICH) as well as patients with favorable clinical outcomes (mRS 0–2) as well as (Figure 2).

### Subgroup-Analysis for Predictors of SICH After Successful ET

The diagnostic performance of NWU, ASPECTS, collateral score, and NIHHS as potential predictors for ICH and also SICH were evaluated by univariable ROC analysis. Comparison of variables for both ICH and SICH displayed the same cut-off values with NWU above 8%, collateral score below 1, NIHSS above 16, and ASPECTS below eight. Detailed results are found in the (Supplemental Table 5, Supplemental Figure 2).

## Discussion

The main finding of our study is that elevated NWU, low collateral score, and low ASPECTS are independent reliable predictors for the development of ICH after successful ET.

In our study the occurrence of SICH after ET was 6.5% and slightly higher compared to 4.4% reported by Goyal et al in the HERMES meta-analysis (1). A reason for the higher incidence of SICH in our study might be due to the higher median age of 79 years, lowers ASPECTS and higher admission serum glucose (Table 1) compared to the HERMES study (median age of 68 years, ASPECTS of 9 and a admission serum glucose of 6.6 mmol/L [118.92 mg/dL]), as they are known significant risk factors for SICH after ET (13). Taking a closer look at the HERMES study, the included SWIFT PRIME and ESCAPE study used threshold of ASPECTS ≤5 and in REVASCAT of ASPECTS <7 on CT as an exclusion criterion for ET (14). In our study no threshold of ASPECTS at baseline were performed for patient inclusion and also included patients with ASPECTS <5 (*n* = 15). In accordance, the MR CLEAN investigators who used the most broadly defined inclusion criteria, reported an SICH incidence of 7.7% in the trial intervention arm (15). It seems also reasonable to consider the different definitions of SICH for the depicted discrepancy in SICH rates. The recently published Heidelberg Bleeding Classification may provide a reasonable solution for this issue.

Pathophysiologically, ICH after ET occurs due to a reperfusion syndrome from rapture of necrotic vessel walls and increased blood-brain barrier permeability due to prolonged ischemia (16). Therefore, ICH due to a reperfusion syndrome is relevant to the degree and time of the reperfusion flow, the time of recanalization and the pretreatment infarct volume (5, 17). In line with this, our results displayed an independent association between lower ASPECTS and the risk of ICH, confirming prior studies (18–20). Not only absolute volume of infarct tissue, but also edema volume on admission CT has been related to the presence of ICH in reperfusion therapy of AIS (17). The severity of brain ischemia can be precisely quantified with a direct CT imaging approach that stratifies early infarct not only by size but also by edema dynamics: Early Net Water Uptake or NWU is a biomarker for the percentage of edematous tissue expansion within infarct lesions. Quantification of water uptake has been first described as an imaging biomarker in AIS to identify patients within thrombolysis time window (in the setting of wake-up strokes) (21). There is growing evidence for the diagnostic importance of NWU as it has also been described as a predictor of malignant infarctions (22). In this study, admission NWU > 12.7% identified malignant infarctions with high discriminative power. In contrast the present study identified NWU > 8% as the best cut-off to predict ICH. The differences in the cut-off for NWU might be caused by the different outcome parameter (malignant infarction vs. ICH), and also by the fact that our study carefully selected only patients with successful ET. The association of water uptake with time demonstrated that NWU increases non-linearly over time (21). In conclusion, the higher cut-off in the study of Broocks et al. can be additionally explained by a prolonged median time from onset to admission imaging of 3.3 vs. 2.4 h in the present study (22). Collateral status, admission ASPECTS, and parenchymal hemorrhagic transformation have displayed a significant association with cerebral edema on CT at 24 h (23). But neither quantitative nor early measurement of cerebral edema has been performed in this study. Ultimately, the relationship of early ischemic changes with respect to occurrence of ICH after successful ET remains unknown. In our study, high NWU was a stronger predictor for ICH after successful ET compared to the other two independent predictors ASPECTS and collateral circulation.

The angiographic grade of collateral flow on admission imaging is not only associated with lower ASPECTS, but also strongly influences the rate of ICH after ET (24–26). Especially, the presence of partial or poor collaterals is related to a higher incidence of bleeding events (24, 25). In line with this, our results demonstrate that partial and in particular poor collaterals are associated with a higher risk of a bleeding event after ET: Patients with poor collaterals had less variances in the probability of a bleeding event, regardless of infarct size (ASPECTS), and edema expansion (NWU). Based on our multivariable analysis, a patient with poor collaterals, and NWU of 15% had an over 95% risk of a bleeding event, regardless of the ASPECT Score (Figure 1). One potential mechanism for early collateral failure is ischemic-induced capillary dysfunction. According to this mechanism, cerebral edema causes elevated interstitial pressure, which increases the resistance of collateral arterioles, downstream perforating arterioles, and capillary beds in the most hypoperfused penumbral zones (23, 26). The diagnostic importance of NWU is highlighted by an illustrative patient with good collaterals and NWU of 15% who has an almost 40-times higher probability of a bleeding event, compared to a patient with only 5% NWU (Figure 1). With an ASPECTS ≤7 the risk even increases almost a 100-fold. Reaffirmation of the clinical relevance of these exemplarily selected values for NWU of 5 and 15%, is given by the very similar medians for NWU in our patient cohort (Table 1).

As particularly SICH poses a major safety concern for ET of AIS, subgroup analysis for SICH was performed (5, 18). In our study only patients with PH type 2 (PHT 2) were classified with SICH. Although only a relatively small number of patients were classified with SICH, our results hold importance as only PHT 2 has been found to independently cause clinical deterioration and impair prognosis (4). ROC analysis revealed same cut-off values for NWU, ASPECTS, and collateral score when performed for prediction of both ICH and SICH, although specificities were overall slightly less for NWU for prediction of SICH compared to ICH.

Nevertheless, our study included all patient with ICH as recent clinical studies call attention to the negative impact of AICH on functional outcome after ET, despite the initial labeling (6, 27). In line with this, a new meta-analysis revealed that ET increases the risk of any ICH compared with medical treatment, when also including the increasing rates of AICH after ET (5). In our study, a proportion of the cases of AICH (15%) after ET were subarachnoid hemorrhage. Ultimately, AICH may undermine long-term neurological functions, although remaining asymptomatic at the time of detection. Neural toxic effects of blood cell disintegration may emerge or deteriorate days or weeks later (28). In this context, in our study patients with ICH received more often intravenous thrombolysis prior to ET by trend. Hence, whilst the difference is only described by trend, the effect of intravenous thrombolysis on the occurrence of AICH does not remain without merit—not least because of the supporting literature (29, 30). Further studies are needed to provide more detailed evidence in order discuss the influence of intravenous thrombolysis on the occurrence of a bleeding event after ET more critically.

To our knowledge, this is the first study to investigate the association of NWU, ASPECTS, and collateral score on the risk of a bleeding event after ET. Imaging examinations may influence the rates of SICH after ET as the incidence of SICH in MR CLEAN was higher than that reported in REVASCAT and ESCAPE which both excluded patients with large ischemic core (1, 14). Our prediction model depicts a good predictive ability for the risk of ICH in patients with AIS treated with successful ET in a real-world practice. The model may aid clinicians to identify patients with the highest as well as the lowest risk of ICH, especially in patients with low ASPECTS (31). However, we cannot propose withholding treatment with ET in patients otherwise eligible according to current guidelines. To address this, future studies using a scoring system with NWU, collateral score and ASPECTS would be conceivable. An external validation of our model is warranted. Future studies should consider using uniformed definitions and standardized procedures in diagnosing ICH.

Some limitations of the present study should be addressed when interpreting the results. Limitations of our study include the retrospective study design and only a relatively small number of patients were recruited, which might lead to false- positive results. As for the accuracy of CT-based NWU measurements, ROIs were drawn by a quantitative edge-detection tool and boundaries adjusted manually if necessary. However, semiautomatic methods of Hounsfield Unit value thresholding have been used in previous studies and partially mitigate a method bias (22, 32). Another point of criticism is the strict inclusion of patients with terminal ICA and MCA occlusion to obtain a homogenous patient cohort and to avoid bias. Further, different thrombectomy devices were used in our study. The types of endovascular device may influence the risk of ICH.

## Data Availability

The data that support the findings of this study are available from the corresponding author upon reasonable request.

## Ethics Statement

This single center retrospective study was approved by the ethics committee (Ethik-Kommission der Ärztekammer Hamburg,WF-018/15) and written informed consent was waived. All study protocols and procedures were conducted in accordance with the Declaration of Helsinki. The data that support the findings of this study are available from the corresponding author upon reasonable request.

## Author Contributions

JN: Study design, acquisition of data, image processing, data analysis, statistical analysis, and drafting the manuscript and revising it critically. HK: Data analysis and drafting the manuscript and revising it critically. GS: Data analysis, statistical analysis, and drafting the manuscript and revising it critically. FF: Acquisition of data and drafting the manuscript and revising it critically. HL: Acquisition of data and drafting the manuscript and revising it critically. RK: Acquisition of data. PS: Data analysis and drafting the manuscript and revising it critically. AK: Study design and drafting the manuscript and revising it critically. GT: Study design and drafting the manuscript and revising it critically. JF: Study design and drafting the manuscript and revising it critically. GB: Study design, acquisition of data, image processing, data analysis, statistical analysis, and drafting the manuscript and revising it critically. UH: Study design, acquisition of data, image processing, data analysis, statistical analysis, and drafting the manuscript and revising it critically.

### Conflict of Interest Statement

JF: Consultant for Acandis, Boehringer Ingelheim, Codman, Microvention, Sequent, Stryker. Speaker for Bayer Healthcare, Bracco, Covidien/ev3, Penumbra, Philips, Siemens. Grants from Bundesministeriums für Wirtschaft und Energie (BMWi), Bundesministerium für Bildung und Forschung (BMBF), Deutsche Forschungsgemeinschaft (DFG), European Union (EU), Covidien, Stryker (THRILL study), Microvention (ERASER study), Philips. GT: Consultant or speaker for Acandis, Bayer Healthcare, Boehringer Ingelheim, BristolMyersSquibb/Pfizer, Covidien, Glaxo Smith Kline; lead investigator of the WAKE-UP study; Principal Investigator of the THRILL study; Grants by the European Union (Grant No. 278276 und 634809) and Deutsche Forschungsgemeinschaft (SFB 936, Projekt C2). AK: Research collaboration agreement: Siemens Healthcare. The remaining authors declare that the research was conducted in the absence of any commercial or financial relationships that could be construed as a potential conflict of interest.
