# Supplementary material for: Hemorrhage After Endovascular Recanalization in Acute Stroke: Lesion Extent, Collaterals and Degree of Ischemic Water Uptake Mediate Tissue Vulnerability
Source: Front Neurol. 2019 Jun 4;10:569. doi: 10.3389/fneur.2019.00569 (PMC6558047; doi:10.3389/fneur.2019.00569)
Supplement: Supplementary file 1 [file Data_Sheet_1.docx]

**Supplemental Material**

**Classification of bleeding events according to Heidelberg Bleeding Classification**

Classification of intracranial hemorrhage (ICH) and relatedness of neurological deterioration and imaging findings was performed according to the Heidelberg Bleeding Classification into the following subtypes ^7^: Hemorrhagic infarction (HI) 1: scattered small petechiae, no mass effect; HI 2: confluent petechiae, no mass effect; parenchymal hematoma (PH) 1: hematoma within infarct tissue and occupied <30% of the infarct volume, no substantive mass effect; PH2: hematoma occupied ≥30% of the infarct volume, with obvious mass effect; remote PH: PH remote from the infarct tissue; intraventricular hemorrhage; subarachnoid hemorrhage; subdural hemorrhage. SICH was diagnosed if the new observed ICH was associated with any of the following conditions: (1) NIHSS score increased >4 points; (2) NIHSS score increased >2 points in a NIHSS; (3) deterioration led to intubation, hemicraniectomy, external ventricular drain placement, or any other medical/surgical intervention. In addition, the symptom deteriorations could not be explained by alternative causes for deterioration other than the observed ICH. Asymptomatic ICH was diagnosed if the new observed ICH was not accompanied by any of the above conditions. Further, the relatedness of neurological deterioration and imaging findings was established.

**Establishing the relatedness of deterioration and imaging findings according to the Heidelberg Bleeding Classification ^7^**

In patients with neurological deterioration the relationship between clinical deterioration and the symptomatic hemorrhagic event is:

1. **Definite:** If any intracranial hemorrhage is the dominant brain pathology on imaging causal for deterioration

2. **Probable:** In the presence of PH2, even if the ischemic infarct may have contributed to deterioration.

3. **Possible:** In the presence of HI2, PH1, rPH, IVH, SAH, SDH, even if ischemic infarct may have contributed predominantly to the deterioration

4. **Unlikely:** In the presence of HI

**Supplemental Table I**

*Title*: Establishing the relatedness of deterioration and imaging findings according to the Heidelberg Bleeding Classification (von Kummer R et al. The Heidelberg Bleeding Classification. *Stroke,* 2015).

| **Definite** | **7** | **PH2 with hemicraniectomy: 3**  **PH2 with death: 3**  **PH2 with incline in NIHSS incline >4: 1** | **hemorrhage shall be classified as symptomatic** | **SICH: 7** |
| --- | --- | --- | --- | --- |
| **Probable** | **0** |  |  |  |
| **Possible** | **3** | **HI2: 2**  **SAH (due to vessel perforation and leading to EVD placement): 1** | **hemorrhage shall be classified as asymptomatic** | **aSICH: 12** |
| **Unlikely** | **9** | **HI1: 5**  **SAH (circumscribed): 4** |  |  |

*Legend:* ICH, intracranial hemorrhagic; HI1, Hemorrhagic infarction 1; HI2, Hemorrhagic infarction 2; PH1, Parenchymal hematoma 1; PH2, Parenchymal hematoma 2; rPH, Remote parenchymal hematoma; IVH, Intraventricular hemorrhage; SAH, Subarachnoid hemorrhage; SDH, subdural hemorrhage, aSICH indicates asymptomatic intracranial hemorrhage and SICH symptomatic intracranial hemorrhage.

*****= SICH: new intracranial hemorrhage detected by brain imaging associated with any of the item below:

1. ≥4 points total NIHSS at the time of diagnosis compared to immediately before worsening.

2. ≥2 point in one NIHSS category. The rationale for this is to capture new hemorrhages that produce new neurological symptoms, making them clearly symptomatic but not causing worsening in the original stroke territory.

3. Leading to intubation/hemicraniectomy/EVD placement or other major medical/surgical intervention.

4. Absence of alternative explanation for deterioration

**Supplemental Table II**

*Title*: Classification of bleeding events after ischemic stroke according to the Heidelberg Bleeding Classification ^7^.

| Heidelberg Bleeding Classification | ICH  (n=37) |
| --- | --- |
| HI 1, n (%) | 6/107 (5.6) |
| HI 2, n (%) | 7/107 (6.5) |
| PH 1, n (%) | 4/107 (3.7) |
| PH 2, n (%) | 7/107 (6.5) |
| rPH, n (%) | 2/107 (1.9) |
| IVH, n (%) | 4/107 (3.7) |
| SAH, n (%) | 16/107 (15.0) |
| SDH, n (%) | 5/107 (4.7) |
| SICH, n (%) | 7/107 (6.5) |
| AICH, n (%) | 30/107 (28.0) |

*Legend:* ICH indicates intracranial hemorrhagic; HI1, Hemorrhagic infarction 1; HI2, Hemorrhagic infarction 2; PH1, Parenchymal hematoma 1; PH2, Parenchymal hematoma 2; rPH, Remote parenchymal hematoma; IVH, Intraventricular hemorrhage; SAH, Subarachnoid hemorrhage; SDH, subdural hemorrhage; AICH, asymptomatic intracranial hemorrhage and SICH, symptomatic intracranial hemorrhage.

**Supplemental Table III**

*Title*: Univariable Analysis of Predictors of intracerebral hemorrhage after recanalization

|  | OR | 95%CI | *P-*Value |
| --- | --- | --- | --- |
| Age at admission [years] | 1.03 | 0.19-1.07 | 0.075 |
| Gender (ref: female) | 0.72 | 0.32-1.60 | 0.421 |
| Hypertension | 1.74 | 0.77-3.97 | 0.185 |
| Diabetes mellitus | 0.77 | 0.27-2.22 | 0.634 |
| Atrial fibrillation | 3.16 | 1.37-7.27 | 0.007 |
| Smoking | 0.78 | 0.24-2.50 | 0.671 |
| Dyslipidemia | 0.79 | 0.29-2.13 | 0.638 |
| Intravenous thrombolysis | 0.62 | 0.28-1.40 | 0.251 |
| NIHSS on admission | 1.10 | 1.01-1.15 | 0.027 |
| ASPECTS | 0.78 | 0.61-1.00 | 0.041 |
| Collateral Score | 0.21 | 0.12-0.40 | <0.001 |
| Net water uptake (NWU) | 1.56 | 1.31-1.86 | <0.001 |

*Legend:* Given for selected variables are Odds Ratios (OR) with 95% confidence interval (CI) and p-value of likelihood ratio test.

**Supplemental Table IV**

*Title*: Multivariable Analysis of Predictors of intracerebral hemorrhage after recanalization

|  | OR | 95%CI | *P-*Value |
| --- | --- | --- | --- |
| Age at admission [years] | - | - | NS: 0.391 |
| Gender (ref: female) | - | - | NS: 0.402 |
| Hypertension | - | - | NS: 0.548 |
| Diabetes mellitus | - | - | NS: 0.973 |
| Atrial fibrillation | - | - | NS: 0.422 |
| Smoking | - | - | NS: 0.693 |
| Dyslipidemia | - | - | NS: 0.133 |
| Intravenous thrombolysis | - | - | NS: 0.766 |
| NIHSS on admission | - | - | NS: 0.417 |
| ASPECTS | 1.90 | 1.07-3.38 | 0.028 |
| Collateral Score | 0.93 | 0.02-0.37 | 0.001 |
| Net water uptake (NWU) | 1.88 | 1.34-2.63 | <0.001 |

*Legend:* Given for selected variables are Odds Ratios (OR) with 95% confidence interval (CI) and p-value of likelihood ratio test.

**Subgroup-analysis**

**Predictors of SICH after successful ET**

To evaluate the diagnostic power of NWU, ASPECTS, collateral score and NIHSS univariable ROC analysis was perfomed (1) for patient with ICH and (2) with SICH. NWU predicted both ICH and SICH with the highest discriminative power with an optimal cut-off above 8% (ICH with an area under the curve [AUC]: 0.90, 95%CI: 0.84-0.96; specificity 74.3%, sensitivity 97.3%, *P*<0.0001 versus SICH with an AUC: 0.78, 95%CI: 0.69-0.85; specificity 55.0%, sensitivity 100%, *P*<0.0001), followed by (2) collateral score with an optimal cut-off below 1 (ICH with an AUC: 0.81, 95%CI: 0.73-0.9; specificity 84.4%, sensitivity 67.6%, *P*<0.0001 versus SICH with an AUC: 0.80, 95%CI: 0.70-0.87; specificity 70.3%, sensitivity 85.7%, *P*<0.0001), (3) NIHSS on admission with an optimal cut-off above 16 (ICH with an AUC: 0.67, 95%CI: 0.56-0.77; specificity 61.4%, sensitivity 64.9%, *P*=0.0026 versus SICH with an AUC: 0.68, 95%CI: 0.57-0.77; specificity 59.0%, sensitivity 71.4%, *P*=0.12) and (4) ASPECTS with an optimal cut-off below 8 (ICH with an AUC: 0.63, 95%CI: 0.52-0.74; specificity 39.1%, sensitivity 86.5%, *P*<0.042 versus SICH with an AUC: 0.51, 95%CI: 0.41-0.61; specificity 68.7%, sensitivity 14.3%, *P*=0.92) (Supplemental Table IV and Supplemental Figure II).

**Supplemental Table V**

*Title:* Receiver operating curve (ROC) analysis for prediction of intracerebral hemorrhage and symptomatic intracerebral hemorrhage after successful endovascular treatment.

| Prediction ICH | AUC | 95% CI | Threshold | Sensitivity | Specificity | *P*-Value |
| --- | --- | --- | --- | --- | --- | --- |
| NWU | 0.90 | 0.84-0.96 | >8 | 97.3 | 74.3 | <0.0001 |
| Collateral Status | 0.81 | 0.73-0.90 | ≤1 | 67.6 | 84.4 | <0.0001 |
| NIHSS on admission | 0.68 | 0,57-0,77 | >17 | 71.4 | 59.0 | 0.0026 |
| ASPECTS | 0.63 | 0.52-0.74 | >8 | 86.5 | 39.1 | 0.042 |
| Prediction SICH | **AUC** | **95% CI** | **Threshold** | **Sensitivity** | **Specificity** | ***P*-Value** |
| NWU | 0.78 | 0.69-0.85 | >8 | 100.0 | 55.0 | <0.0001 |
| Collateral Status | 0.80 | 0.70-0.87 | ≤1 | 85.7 | 70.3 | <0.0001 |
| NIHSS on admission | 0.68 | 0,57-0,77 | >17 | 71.4 | 59.0 | 0.12 |
| ASPECTS | 0.51 | 0.41-0.61 | >8 | 14.3 | 68.7 | 0.92 |

*Legend:*  Alberta Stroke Programme Early CT Score (ASPECTS); Area under the curve (AUC); 95% confidence interval (CI); intracerebral hemorrhage (ICH); National Institutes of Health Stroke Scale (NIHSS); Net Water Uptake (NWU); symptomatic intracerebral hemorrhage (ICH).

**Supplemental Figure I**

*Title:* Patient enrollment

^*1^


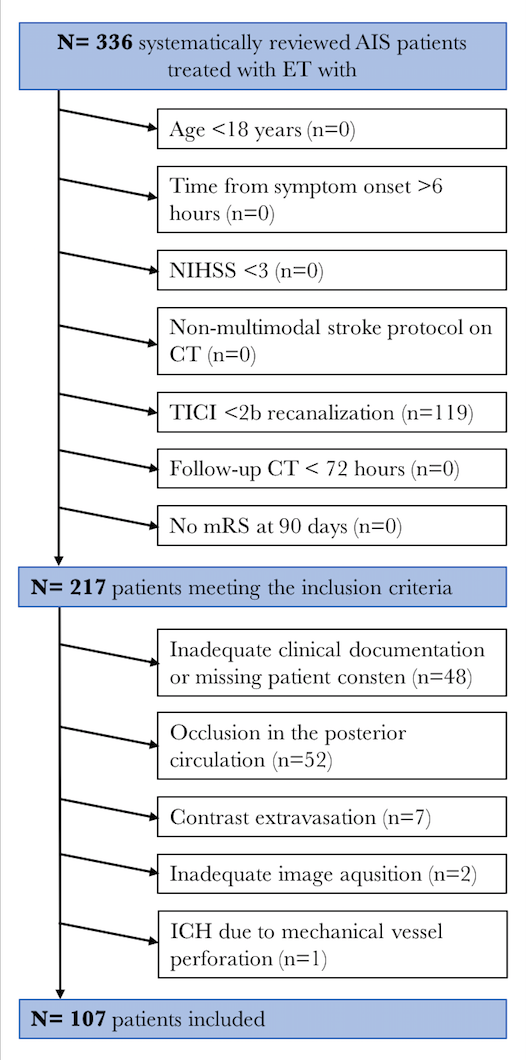


Patient enrollment according to inclusion and exclusion criteria with absence of a preexisting thromboembolic or hemodynamic infarction in admission non-enhanced CT. Hyperdense phenomena without mass effect on CT and disappearance with 24 hours on follow-up CT were classified as contrast extravasation ^12^.

*Legend:* CT, Computed Tomography; ICH, intracranial hemorrhagic; NIHSS, National Institutes of Health Stroke Scale; TICI, thrombolysis in cerebral infarction.

**Supplemental Figure II**

*Title:* Receiver operating curve (ROC) analysis for prediction of intracerebral hemorrhage after successful endovascular treatment.


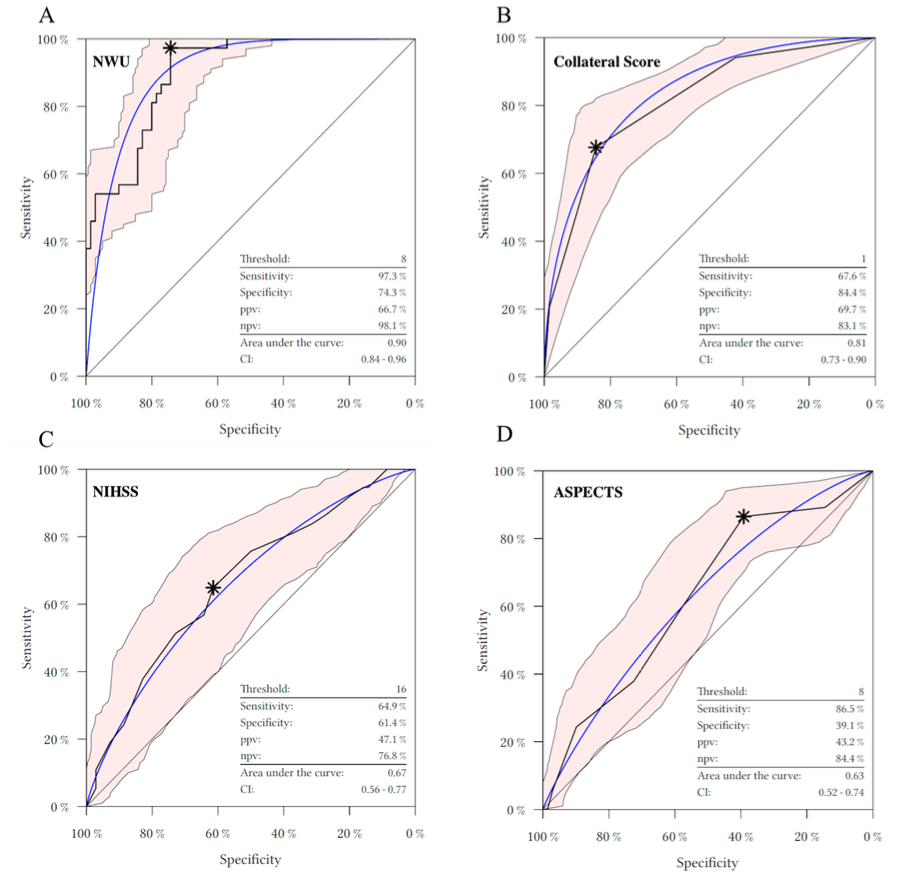


*Legend:*  Alberta Stroke Programme Early CT Score (ASPECTS); Area under the curve (AUC); 95% confidence interval (CI); National Institutes of Health Stroke Scale (NIHSS); negative predictive value (npv); Net Water Uptake (NWU); positive predictive value (ppv).
